# A comparative investigation of machine learning algorithms for predicting safety signs comprehension based on socio-demographic factors and cognitive sign features

**DOI:** 10.1038/s41598-023-38065-1

**Published:** 2023-07-05

**Authors:** Sajjad Rostamzadeh, Alireza Abouhossein, Mahnaz Saremi, Fereshteh Taheri, Mobin Ebrahimian, Shahram Vosoughi

**Affiliations:** 1 Department of Ergonomics, School of Public Health and Safety, Shahid Beheshti University of Medical Sciences, Tehran, Iran; 2 Occupational Health Research Center, Iran University of Medical Sciences, Shahid Hemmat Highway, Tehran, 1449614535 Iran; 3 Department of Health in Disasters and Emergencies, University of Social Welfare and Rehabilitation Sciences, Tehran, Iran

**Keywords:** Psychology, Health occupations

## Abstract

This study examines whether the socio-demographic factors and cognitive sign features can be used for envisaging safety signs comprehensibility using predictive machine learning (ML) techniques. This study will determine the role of different machine learning components such as feature selection and classification to determine suitable factors for safety construction signs comprehensibility. A total of 2310 participants were requested to guess the meaning of 20 construction safety signs (four items for each of the mandatory, prohibition, emergency, warning, and firefighting signs) using the open-ended method. Moreover, the participants were asked to rate the cognitive design features of each sign in terms of familiarity, concreteness, simplicity, meaningfulness, and semantic closeness on a 0–100 rating scale. Subsequently, all eight features (age, experience, education level, familiarity, concreteness, meaningfulness, semantic closeness, and simplicity) were used for classification. Furthermore, the 14 most popular supervised classifiers were implemented and evaluated for safety sign comprehensibility prediction using these eight features. Also, filter and wrapper methods were used as feature selection techniques. Results of feature selection techniques indicate that among the eight features considered in this study, familiarity, simplicity, and meaningfulness are found to be the most relevant and effective components in predicting the comprehensibility of selected safety signs. Further, when these three features are used for classification, the K-NN classifier achieves the highest classification accuracy of 94.369% followed by medium Gaussian SVM which achieves a classification accuracy of 76.075% under hold-out data division protocol. The machine learning (ML) technique was adopted as a promising approach to addressing the issue of comprehensibility, especially in terms of determining factors affecting the safety signs' comprehension. The cognitive sign features of familiarity, simplicity, and meaningfulness can provide useful information in terms of designing user-friendly safety signs.

## Introduction

Safety signs are part of the safety management systems (SMS) to improve the safety of construction sites by increasing workers' awareness of safety instructions, which helps to regulate, warn, and guide construction workers against occupational risks^1,2^. Safety signs are common tools used to communicate effectively besides or instead of verbal information, as comprehension of the signs can overcome the language barrier^3^. In an attempt to promote safety awareness and reduce human errors, pictorial safety signs can potentially convey large amounts of safety information in minimal space, they can be more effective visual communication than textual signs, and be remembered better than simple text^4,5^. Safety signs can be confusing and misleading if they are similar in shape and color and therefore can be interpreted by the individual with preconceived notions in mind^3^. Recent studies have shown that comprehension varies widely among different safety signs and the effectiveness of some safety signs is low in terms of conveying safety messages^6,7^. Sign comprehensibility is usually measured through a comprehension test, in which comprehension accuracy of over 67.00% is considered acceptable according to the International Organization for Standardization (ISO 3864, 2011)^8^. The American National Standards Institute (ANSI) set a higher acceptable comprehension level of 85.0% (ANSI Z535.3, 1997)^9^. Based on past studies, various socio-demographic factors such as age, gender, education level, cultural differences, working experience, duration of work, training, and type of safety signs affect the safety signs' comprehensibility. With particular regard to cultural background, Caffaro et al. and Yao et al. highlighted that the same signs may have different meanings in different cultures^10,11^. This consideration points out an important issue in terms of occupational safety and risk communication, especially in light of the increasing cultural diversity among construction workers who often migrated to work in countries different from their own^6^. Some studies have also found that the sign comprehension time increased with participant age and the number of information units and was shorter for male counterparts^12,13^. Ben-Bassat et al. and Jiang et al. showed that older adults may have particular difficulty in understanding high complexity and low comprehensibility signs due developing physical and cognitive constraints^14,15^. Similarly, Gao et al. addressed work experience and education level as significant predictors of sign comprehension among workforces^16^. They stated that the subjects with the relevant workplace or site visit experiences were usually more familiar with the context for safety signs and thus performed better in sign comprehension. In addition to the qualitative findings on the importance of socio-demographic factors for safety signs comprehension discussed above, Patel et al. performed a quantitative analysis to find out whether cognitive sign features influenced the comprehensibility of the safety signs^17^. These features, previously formulated by McDougall et al. included familiarity (i.e. the frequency of encountering a sign), concreteness (i.e. the sign depicts real objects, materials, or people), simplicity (i.e. the sign has a low number of elements, and detail), meaningfulness (i.e. subjects can attach a meaning to a sign), and semantic distance (i.e. the closeness of the relationship between the graphic and the desired function)^18^.

Previous studies have been conducted to gain a better understanding of the pattern of safety signs comprehensibility. The findings were chiefly limited to determining the signs comprehension scores and in some cases finding factors affecting comprehensibility by conventional descriptive statistics^7,19^, analysis of variance^6,20^, and multiple regression^2^. Many input features, incorrect segmentation, and irrelevant features in these studies often make a predictive modelling task more challenging and it leads to less reliable predictive models. Unlike traditional statistical methods that are aimed at inferring relationships between variables, Machine Learning (ML) concentrates on making predictions as accurately as possible by using general-purpose learning algorithms^21^. Many efforts have been made using ML approaches to identify different contributing factors in accidents, diseases, and injuries occurrence. Gilani et al. used the ML methods such as artificial neural network (ANN) and logistic regression models to identify the influential factors on urban traffic accident occurrence. The results of this research indicated that the ANN model was more able, in terms of accuracy and efficacy, to predict the severity of accidents^22^. Ganggayah et al. utilized six types of machine learning algorithms, which included decision tree (DT), random forest (RF), neural networks (NN), extreme boost, binary logistic regression (BLR), and support vector machine (SVM), to build models for detecting and visualizing significant prognostic indicators of breast cancer survival rate. The results showed that random forest (RF) and decision tree (DT) had the highest and lowest accuracy with 82.7% and 79.8%, respectively^23^.

Despite the wide variety of applications of the ML approach in various research fields of bioinformatics and diagnostics, the ability of different ML algorithms in predicting different psychological and cognitive components and how those components affect the decision-making outcome has received less attention. The majority of the studies about safety signs mainly examine the person’s psychological and neurophysiological aspects and the compliance behaviors that are directly in response to the safety signs^24–26^. Their subjective impressions and evaluations are often influenced by the demographic characteristics of the person, such as gender, work experience, education level, and age group. These influencing factors can manifest in partial systematic biases, and cause serious challenges in achieving a common view reducing comprehensibility results that are less repeatable and less accurate in predicting the dependent variable(s). To our knowledge, the current study can be considered a pioneer in the field of cognitive ergonomics and safety signs comprehensibility by quantifying and evaluating the effectiveness and capabilities of different supervised ML algorithms in predicting the safety signs comprehensibility and determining its most important predictors. The ML approach can provide an integrated view of trends in construction workers' comprehensibility and behavioral patterns, eliminate the biases in people's understanding of a certain subject, and supports the development and design of new safety signs. Based on this, we used a numerical approach presented in the table instead of its graphical representation for a comparative investigation of ML algorithms to show its applicability and easier understanding for researchers and readers in other scientific fields. The objectives of the study were three-fold: (a) investigate the feasibility of socio-demographic factors and cognitive sign features in safety signs comprehensibility prediction, (b) quantify, implement, evaluate, and analyze the performance of the 14 different ML models for comprehensibility prediction in construction safety signs, and (c) which of the ML algorithms can predict the safety signs comprehensibility with the highest accuracy and consistency.

## Materials and methods

### Study area

The study was carried out between April and October 2021 among construction sites in Tehran City, Iran. Tehran is the capital city of Iran with over 15 million people dwellings in the larger metropolitan area of Tehran province^27,28^. As a large metropolitan city that has a pivotal attribute to the political and financial part of Iran, it attracts many skilled and low-skilled workers from inside and neighboring counties to work in the ongoing construction projects of the city.

### Subjects and sampling

The study population comprised 2310 male construction workers between the age of 18 and 63 years from different districts of the major metropolitan city of Tehran, Iran. The three-stage sampling method was utilized. At first, a stratified sampling method was used to identify five clusters based on population distribution in Tehran. In the second stage, after providing the list of all the construction projects located in selected clusters, a systematic random sampling method was applied to choose five construction projects per cluster. The required minimum sample size of 400 subjects in each cluster (80 for each construction project) was determined using the formula,

$$ n = \, \left( {{\text{Z}}_{{{1} - \alpha /{2}}} + {\text{Z}}_{{{1} - \beta }} } \right)^{{2}} {\text{s}}^{{2}} /{\text{d}}^{{2}} , $$

where Z_1−α/2_ = 1.96 (the value of normal deviate at 0.05 level of confidence), Z_1−β_ = 0.85 (the value of normal deviate at the study power of 0.8), d = 2.4 (expected absolute allowable error in the mean), and s = expected standard deviation of 17.1 according to the study conducted by Chan et al.^29^. Considering the “Design effect” for clustered sampling method (Deff = 2.2)^30^, the desired sample size was obtained to be 2310 subjects with about a 10% non-response rate.

All participants were Persian-speaking with self-declared normal or corrected-to-normal vision and good mental and physical health status at the survey time. Those who disagreed to participate had blurred or poor vision and diabetes and were not enrolled in the study. Participants were given information on what the study was about. Informed consent was obtained from all subjects and/or their legal guardian(s). Participants were assured of the complete confidentiality of the study and data and results were kept secured based on local instructions of the University for Data Protection Act. The study protocol was approved by the Research and Ethics Committee of the Iran University of Medical Sciences (Reg. IR.IUMS.REC.1397.177). All methods were performed following relevant guidelines and regulations.

### Safety sign selection

**Figure 1:**
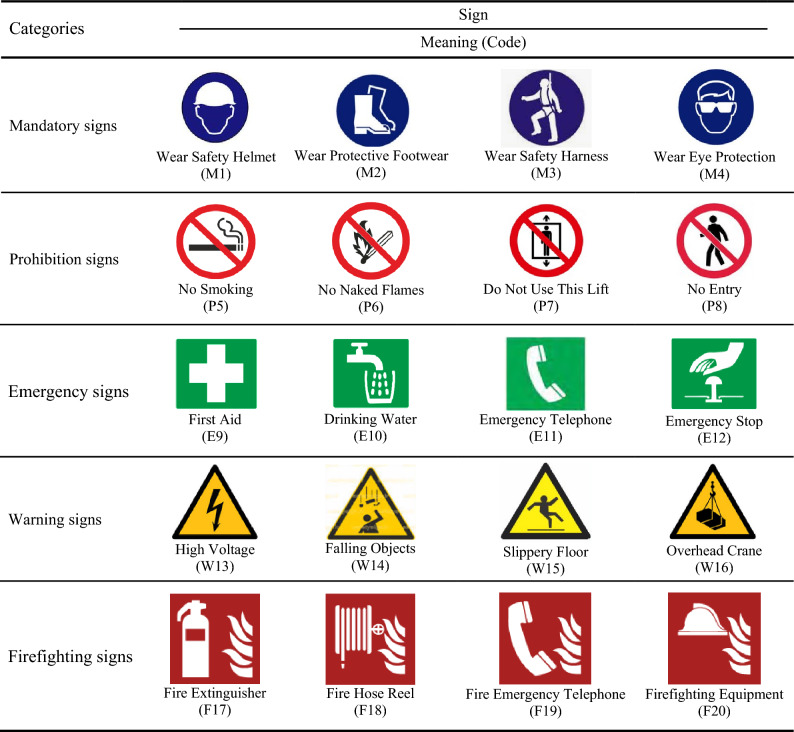
Safety signs used in the study and their intended meanings (from ISO 3864-2:2016).

Safety signs for a varied range of hazard types were included to foster greater generalizability of the test results. 15 health and safety experts participated in the safety signs selection process. The experts were identified and selected according to the snowball technique (also known as chain-referral sampling), which is a non-probability (non-random) sampling method used when characteristics to be possessed by samples are rare and difficult to find^31^. To select the safety signs, all 220 safety signs of the ISO 3864-2:2016 standard^32^ (42 mandatory signs, 42 prohibition signs, 50 emergency signs, 55 warning signs, and 31 firefighting signs) were printed color in squares of 2 × 2 cm on separate white papers. Then, these signs were sent to safety experts, and they were asked to select in such a way that are infrequently used and have a certain type and purpose in all five categories including mandatory, prohibition, emergency, warning, and firefighting. Finally, 20 safety signs including 4 mandatory signs (with code M1–M4), 4 prohibition signs (with code P1–P4), 4 emergency signs (with code E1–E4), 4 warning signs (with code W1–W4), and 4 firefighting signs (with code F1–F4) were selected. Figure 1 shows the final set of safety signs with their code and their respective intended meanings.

### Experimental design and procedure

The data were collected using a questionnaire with three sections in the native language of the participants (Persian). In the process of designing the questionnaire, contributions of industrial and organizational psychologists, health and safety specialists, civil engineers, and enforcement agencies resulted in a construction characteristics portion and a construction safety signs evaluation portion.

#### Socio-demographic characteristics

The first part comprised questions including age, education level, years of experience, occupational status, and previous sign-related knowledge. Since subjects' prior knowledge and experience could affect the results of the study, these people were excluded.

#### Safety signs comprehensibility

For the evaluation portion of the construction safety sign (second part), 20 signs were printed as color photographs (approximately 7 $\times$ 7 cm in size) on a separate sheet of A4 white paper (correct meanings were not included). The papers were evenly assigned to 10 test booklets, within 20 non-duplicated safety signs. Each participant responded to only one test booklet randomly attributed to him. The basic method of assessment was open-comprehension testing as described in ANSI (American National Standards Institute) Z535.3 (2007b)^33^ and ISO (International Organization for Standardization) 9186 (2001)^34^. The examiner verbally asked the participant the following questions: (1) Have you ever seen this sign? (2) What is the meaning of this safety sign? (3) What should be done when this safety sign is seen? In addition to the verbal questioning, the questions were also printed on sheets that each participant could read at the same time. This procedure was suggested by ISO 9186 (2001) and was thus used in determining the comprehension correctness level in the present study^34^. Participants were tested individually and gave oral answers for the entire experimental procedure.

Comprehension data were obtained separately for the pictorial signs and the signs’ background color and shape code. Authors with other two graphic/communication design experts’ judges individually scored all participant responses. While doing the scoring, the judges had each symbol’s intended meaning and the participant’s written responses. Their task was to decide, independently, whether the participants’ interpretations were matching to the intended meanings of signs by assigning a score of “1” to correct responses and a score of “0” to incorrect ones. If the three judges were unable to agree on the judgment for a response, consensus-based decision makings were used. To ensure the reliability of this process, inter-rater reliability was calculated by averaging the amount of accordance between judges, which reached 94.7%. Correctness of comprehension of the meaning of the safety sign was determined based on the following seven standard categories suggested by ISO 9186 (2001):

1. A correct comprehension of the sign meaning is certain (estimated probability of correct understanding over 80%).
2. A correct comprehension of the sign meaning is very probable (estimated probability of correct understanding between 66 and 80%).
3. A correct comprehension of the sign meaning is probable (estimated probability of correct understanding between 50 and 65%).
4. The meaning, which is understood, is opposite to that intended.
5. Any other response.
6. The response given is “don’t know”.
7. No response is given.

Then, the percentage of participants’ responses obtained in the first three categories was multiplied by a factor of correction, described in ISO 9186 (2001), as follows:

1. The percentage is multiplied by 1, if the correct understanding is certain.
2. The percentage is multiplied by 0.75, if correct understanding is very probable.
3. The percentage is multiplied by 0.5, if a correct understanding is probable.

The sum of these three values was labeled as “Score”. The percentage of responses classified as the opposite (category 4) was subtracted from the “Score” resulting in the “Overall Score”. The presence of negative scores is explained by the existence of high percentages of opposite meanings that were generated (i.e., critical confusion).

A criterion used for sign comprehension testing was adapted to fit the role of measuring participants’ interpretation of the shape-color background meaning (separate from the sign). The shape–color code was assessed relative to the following:

- Mandatory: round shape, a white symbol on blue background.
- Prohibition: round shape, a black symbol on white background, red edging, and diagonal line.
- Emergency: square or rectangular shape, a white symbol on a green background.
- Firefighting: square or rectangular shape, a white symbol on a red background.
- Warning: equilateral triangle shape, a black band with a black symbol on yellow background.

This evaluation was performed from the answers given to the question “What do you think the sign means?” Completely correct responses should include the meaning of the symbol and the shape–color code. Critical confusion were assessed by responses attributing the opposite meaning to the shape and color components. To this purpose, participants’ answers to the question “What action would you take in response to this safety sign?” were evaluated. The criterion for safety sign acceptance is at least 85% of test subjects correctly interpret the icon/ pictogram and no more than 5% of subjects are critically confused, based on the ANSI Z535.3 recommendations^33^. Also, ISO 3864 was used as a similar comprehension criterion for safety signs with a minimum correct recognition rate of 66.7%^32^.

#### Cognitive sign features

In the third section, the cognitive sign features test was provided to record subjects' viewpoints about each construction safety sign, proposed by Mcdougall et al.^18^. The authors reported strong validity and reliabilities for the original version, leading several researchers to use it thereafter^35,36^. The Persian version of this questionnaire, validated by Taheri et al. (2018), was applied in the present study^37^. The cognitive sign features sheets considered five features namely familiarity, concreteness, simplicity, meaningfulness, and semantic closeness. Familiarity refers to the rate at which a sign has ever been encountered. Signs are considered concrete if they are drawn similarly to real objects. The criterion of simplicity indicates the degree to which the signs are detailed. Meaningfulness indicates how meaningful users perceive a sign. Semantic closeness refers to the closeness of the association between what is depicted on a sign and what it is intended to represent. Complete explanations about the meaning of the five cognitive sign features and the rating instructions were given to each participant. Participants were requested to subjectively rate the design features for each safety sign on a 0–100 point scale for familiarity (0 = *very unfamiliar,* 100 = *very familiar*), concreteness (0 = *clearly abstract,* 100 = *clearly concrete*), simplicity (0 = *very complex,* 100 = *very simple*), meaningfulness (0 = *completely meaningless,* 100 = *completely meaningful*), and accuracy of semantic closeness (0 = very *weakly related,* 100 = very *strongly related*). The ratings were marked on 5-item questionnaires embedded under the given sign on each page of the test booklet (described above). The total time to complete a test booklet took about 30–45 min for each participant. The process was repeated until all safety signs were completely rated. The entire interview process was guided by a sole investigator (the second author). The local research ethics committee approved the study protocol.

### Descriptive analysis

Statistical analysis was performed by SPSS 23 (IBM Corporation, New York, NY, United States). The normality test was carried out using the Kolmogorov–Smirnov test for all data sets. Statistical outliers were checked using the Grubb′s test which is based on the difference between the mean of the sample and the most extreme data considering the standard deviation^38^. Relative and absolute reliability was assessed for the comprehension performance test using the Intra-class Correlation Coefficient (ICC) and standard error of the measurement (SEM), respectively. Basic descriptive statistics such as means, frequencies, and percentages were calculated for both demographic characteristics as well as cognitive sign features and comprehension performance scores. An analysis of covariance (ANCOVA) with Bonferroni-adjusted post-hoc tests was then performed to test the effects of socio-demographic factors and cognitive sign features included in the study on the comprehension rate.

### Statistical learning approach

**Figure 2:**
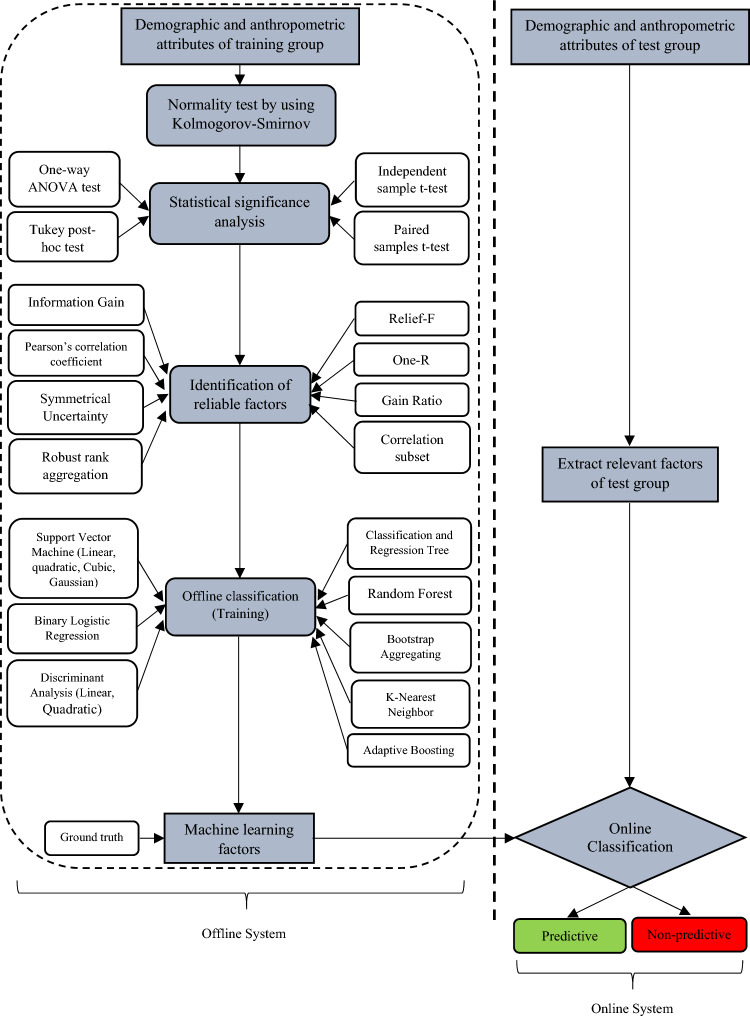
Proposed strategy for prediction of safety signs comprehensibility.

The proposed archetype for the prediction of safety signs comprehensibility using socio-demographic and cognitive signs features in the ML approach is presented in Fig. 2. The left side and right side of Fig. 2 show the offline system (training phase) and online mode (testing phase), respectively. The implementation steps of these phases are explained in the following sections along with details of the dataset used in this study.

#### Feature selection

The selection of reliable factors plays a crucial role in safety signs comprehensibility representation and classification using machine learning (ML) techniques. Feature selection is a procedure of choosing the most pertinent features and building a sensible model with better prediction power for signs comprehensibility. Broadly, feature selection techniques are classified into two types namely, filter and wrapper methods. Filter methods measure the relevance of features by their correlation with the corresponding variable while wrapper methods attempt to find the “optimal” feature subset by iteratively selecting features based on the classifier performance. In this study, we used filter methods to determine the rank of features and select the relevant features by some principal criteria such as Information Gain (IG)^39^, Pearson’s correlation coefficient (P)^40^, 1R^41^, Gain Ratio (GR)^42^, Relief-F (RF)^43^, and Symmetrical Uncertainty (SU)^44^ in decreasing order. Also, a correlation-based wrapper feature selection (CFS) approach was used to select the most reliable subset of components^45^. This method generates different possible subsets from the given number of features and then evaluates them using a specific objective function. We kept the subset of features with the highest performance and discarded all other subsets. Further, a robust rank aggregation (RRA) technique, as a hybrid approach, was also implemented and evaluated^46^.

#### Classification

The final phase of any ML approach is the classifier which maps input feature vectors x ∈ X to output class labels y ∈ {1,…, n}, where X is the feature space and n is the total number of classes. Classification techniques are broadly classified into two types namely, supervised, and unsupervised. In a supervised classifier, the training samples are supplied along with their class labels. The class label of unknown cases i.e. the test samples is then determined based on the parameters of the trained classifier model. In this study, some of the most popular supervised classifiers such as Binary Logistic Regression (BLR), Linear Discriminant Analysis (LDA), Quadratic Discriminant Analysis (QDA), Classification and Regression Tree (CART), Support Vector Machine (SVM), Random Forest (RF), Bootstrap Aggregating (also known as Bagging) algorithm, K-Nearest Neighbor (K-NN), and Adaptive Boosting were used to predict which of the socio-demographic factors and cognitive sign features (i.e. independent variables) are importance on the safety signs comprehensibility (i.e. dependent variable). We chose these classifiers because, according to the literature, these classifiers have been efficaciously used in previous Computer-Aided Diagnosis (CAD) studies^47–51^. The overall machine learning analysis was programmed using Scikit-Learn 0.20.3, a popular Python ML library^52^.

#### Performance evaluation metrics and methods

The various performance metrics used to evaluate the classifiers are classification sensitivity or recall, specificity, accuracy, precision, F1 score, and area under the curve (AUC)^53^. Sensitivity or recall is the performance of a classifier to correctly categorize a person with correct comprehensibility as a positive class; specificity is the performance of a classifier to correctly categorize a subject with incorrect comprehensibility as a negative class; accuracy is the fraction of the individual who was correctly classified as a positive or negative class by an ML model; precision, also known as a positive predictive value, is the fraction of the true positive class among the workers who were predicted as a positive class; F1 score is the harmonic mean of precision and recall^54^. Along with these performance measures, the area under the receiver operating characteristics (AUC) is also used to compare classifier models. The mathematical formulas to calculate the above performance metrics are shown in Eqs. (1)–(5)^53,55^.

1 $$\mathrm{Sensitivity \,\,or\,\, recall}=\frac{\mathrm{TP}}{\mathrm{TP}+\mathrm{FN}}$$

2 $$\mathrm{Specificity}=\frac{\mathrm{TN}}{\mathrm{TN}+\mathrm{FP}}$$

3 $$\mathrm{Accuracy}=\frac{\mathrm{TP}+\mathrm{TN}}{\mathrm{TP}+\mathrm{TN}+\mathrm{FP}+\mathrm{FN}}$$

4 $$\mathrm{Precision}=\frac{\mathrm{TP}}{\mathrm{TP}+\mathrm{FP}}$$

5 $$\mathrm{F}1=\frac{2\mathrm{TP}}{2\mathrm{TP}+\mathrm{FP}+\mathrm{FN}}$$

where, TP: true positive, FP: false positive, FN: false negative, and TN: true negative.

#### Data division protocol

K-fold cross-validation was used in this study to compare the model performance with that of existing predictors which is the most popular and extensively acknowledged by the research community. In this approach, the whole dataset was divided into 'k' groups, consisting of an approximately equal number of samples. Out of the 'k' groups, 'k − 1' groups are used for training the classifier model while the remaining group is used for testing purposes^56^. The process is repeated 'k' times and average performance over 'k' rounds is calculated. In this study, experiments were conducted with the desired value of k = 10, and the average results were used to evaluate the model^57,58^.

## Results

### Demographic characteristics

The experiment included 2310 construction male workers ranging in age from 18 to 63 years (mean = 45.31, SD = 11.27). All of the participants had at least 5 years and more (between 6 and 45 years) of construction work experience (mean = 16.45, SD = 2.13) and more than half of the total subjects were in the 18–35 age range. The main demographic characteristics of the sample are reported in Table 1.

**Table 1:** Distribution of participants based on their characteristics.

Variable	Category	Frequency	Percent
Gender	Male	2310	100.0
	Female	–	–
Marital status	Single	1408	61.0
	Married	902	39.0
Age group	≤ 25 years	679	29.4
	26–35 years	580	25.1
	36–45 years	460	19.9
	46–55 years	330	14.3
	≥ 56 years	261	11.3
Work experience	5–15 years	1107	47.9
	16–30 years	740	32.1
	31–45 years	463	20.0
Education level	MS/Ph.D	33	1.4
	Bachelor’s degree	456	19.7
	High school	1011	43.8
	Less than high school	810	35.1

### Comprehension score of signs

Table 2 shows the overall scores (mean ± SD) for comprehension of pictorial symbol meaning and shape-color code. “Do not use this lift” (P7) and “No Smoking” (P5) signs had minimum and maximum comprehension of sign meaning (16.6% for P7 and 89.4% for P5) and shape-color code (− 4.1% for P7 and 92.2% for P5), respectively. The American National Standard Institute (ANSI) and Organization for International Standardization (ISO) have recommended that symbols must reach a criterion of at least 85% or 67% correct, respectively, in a comprehension test to be considered acceptable^59^. As shown in Table 2, there was only one safety sign reaching both the ISO and ANSI criteria, “No smoking” (P5; 89.4%). Another seven safety signs achieved the lower criteria of ISO only, namely the “Wear eye protection” (M4; 71.4%), “No naked flames” (P6; 78.8%), “No entry” (P8; 71.8%), “First aid” (E9; 77.7%), “High voltage” (W13; 73.6%), “Fire extinguisher” (F17; 81.2%), “Firefighting equipment” (F20; 67.6%). The overall mean sign comprehension scores across all safety signs for each of the five sign groups were:

- Mandatory signs: 56.13% (SD = 11.24), ranging from − 17.21 (min.) to 88.52 (max.).
- Prohibition signs: 64.15% (SD = 10.21), ranging from − 6.89 (min.) to 91.39 (max.).
- Emergency signs: 57.05% (SD = 8.31), ranging from − 23.50 (min.) to 86.77 (max.).
- Warning signs: 54.22% (SD = 9.51), ranging from − 19.23 (min.) to 89.22 (max.).
- Firefighting signs: 62.00% (SD = 10.44), ranging from − 5.77 (min.) to 94.35 (max.).

**Table 2:** Overall scores (mean ± SD) for comprehension of significant meaning and shape-color for the 20 safety signs.

Code	Sign meaning			Sign shape-color		
	% (Mean ± SD)	Satisfy		% (Mean ± SD)	Satisfy	
		ISO	ANSI		ISO	ANSI
	61.8 ± 7.21			**71.3 ± 8.03**	✓	
	61.4 ± 9.07			**83.4 ± 5.37**	✓	
	29.9 ± 7.89			38.2 ± 8.11		
	**71.4 ± 9.24**	✓		**86.7 ± 8.00**	✓	✓
	**89.4 ± 11.10**	✓	✓	**92.2 ± 7.00**	✓	✓
	**78.8 ± 11.45**	✓		**75.0 ± 10.30**	✓	
	16.6 ± 7.56			− 4.1 ± 3.12		
	**71.8 ± 7.22**	✓		**87.5 ± 10.10**	✓	✓
	**77.7 ± 12.14**	✓		**89.1 ± 11.32**	✓	✓
	65.3 ± 10.91			**70.1 ± 8.14**	✓	
	35.8 ± 5.77			**77.2 ± 8.31**	✓	
	49.4 ± 7.11			47.32 ± 7.21		
	**73.6 ± 8.08**	✓		**71.5 ± 9.10**	✓	
	52.3 ± 8.13			46.4 ± 7.24		
	66.4 ± 8.73			**67.7 ± 9.11**	✓	
	24.6 ± 5.11			48.7 ± 6.65		
	**81.2 ± 8.77**	✓		**88.5 ± 10.73**	✓	✓
	47.9 ± 8.21			52.7 ± 8.40		
	51.3 ± 9.12			**71.2 ± 10.27**	✓	
	**67.6 ± 6.27**	✓		**86.4 ± 7.10**	✓	✓

*M* mandatory signs, *P* prohibition signs, *E* emergency signs, *W* warning signs, *F* firefighting signs, *SD* standard deviation.

Significant values are in bold.

The data show that the prohibition signs attained a somewhat higher level of sign-meaning comprehension than the warning, emergency, and mandatory signs, followed closely by the firefighting signs. The overall mean comprehension for the signs across participants was 59.13% (SD = 16.45), ranging from − 23.50 to 94.35. Friedman's two-way analysis of variance by ranks test revealed that there was a significant effect of sign group in the scores of comprehensions of sign meaning (χ^2^(4) = 17.35, p < 0.001). Dunn–Bonferroni pairwise multiple comparisons indicated that both prohibition signs (Median = 46.3, Interquartile Range (IQR) = 48.6; p = 0.001) and firefighting signs (Median = 52.3, IQR = 55.0; p = 0.001) scored significantly higher than the mandatory signs (Median = 17.8, IQR = 29.8), emergency signs (Median = 18.8, IQR = 38.1), and warning signs (Median = 14.2, IQR = 31.0) groups; whereas there were no significant differences among the mandatory, emergency, and warning sign groups.

### Comprehension score of shape–color code

Comprehension of the safety signs’ shape-color coding was also checked out (see Table 2). The 67% level (similar to the ISO sign comprehension criterion) and the 85% level (similar to the ANSI sign comprehension criterion) were used as standard acceptability criteria to compare to the levels found in the present study. There were seven safety signs reaching both the ISO and ANSI criteria, namely the “Wear eye protection” (M4; 86.7%), “No smoking” (P5; 92.2%), “No entry” (P8; 87.5%), “First aid” (E9; 89.1%), “Fire extinguisher” (F17; 88.5%), and “Firefighting equipment” (F20; 86.4%). Only 14 out of 20 safety signs attained 67% comprehension criterion for shape–color in the present study (signs M1—Wear safety helmet; M2—Wear protective footwear; M4—Wear eye protection; P5—No smoking; P6—No naked flames; P8—No entry; E9—First Aid; E10—Drinking water; E11—Emergency telephone; W13—High voltage; W15—Slippery floor; F17—Firefighting extinguisher; F19—Fire emergency telephone; F20—Firefighting equipment). Table 2 have shown that several instances of the signs’ shape–color coding were poorly comprehended (signs M3—Wear safety harness; P7—Do not use this lift; E12—Emergency stop; W14—Falling objects; W16—Overhead crane; F18—Fire hose reel). The mean shape–color code comprehension scores across all safety signs for each of the five sign groups were:

- Mandatory signs: 70.20% (SD = 10.26), ranging from 6.35 (min.) to 96.33 (max.).
- Prohibition signs: 62.65% (SD = 8.67), ranging from − 18.25 (min.) to 88.09 (max.).
- Emergency signs: 70.93% (SD = 10.54), ranging from − 12.44 (min.) to 92.25 (max.).
- Warning signs: 58.58% (SD = 8.37), ranging from − 16.45 (min.) to 85.71 (max.).
- Firefighting signs: 74.70% (SD = 11.89), ranging from − 8.10 (min.) to 98.21 (max.).

The data show that the firefighting signs attained a somewhat higher level of sign shape–color code comprehension than the warning, emergency, prohibition, and mandatory signs. The overall mean sign shape–color code comprehension across participants was 67.41% (SD = 18.27), ranging from − 18.25 to 98.21. Friedman's two-way analysis of variance by ranks test revealed that there was a significant effect of sign group on the sign shape–color comprehension (χ^2^(4) = 16.10, p < 0.001). Dunn–Bonferroni pairwise multiple comparisons indicated that firefighting signs (Median = 57.4, IQR = 54.6; p = 0.001) scored significantly higher than the mandatory signs (Median = 38.6, IQR = 57.1; p < 0.001), emergency signs (Median = 33.8, IQR = 49.0; p < 0.001), prohibition signs (Median = 27.4, IQR = 45.0; p < 0.001), and warning signs (Median = 29.4, IQR = 32.0; p < 0.001) groups. Also, there was a significant difference between each of the mandatory and emergency signs with prohibition and warning signs; but there were no significant differences between the mandatory and emergency signs.

Table 3 shows that 7 of the 20 signs generated at least some critical confusion (opposite answers). Scores with bold markings in Table 3 show the particular signs that exceeded the ANSI Z535.3 acceptability level of attaining more than 5% critical confusion for comprehension of pictorial symbols and shape–color code. According to ANSI Z535.3, signs that exceed the 5% critical confusion level should be rejected. Based on this, three safety signs would be rejected based on comprehension scores of sign meaning and shape-color. These signs were: M3—“Wear safety harness”, P7—“Do not use this lift”, W14—“falling objects”. Generally, the workers had the largest number of critical confusions for shape-color comprehension of signs than comprehension of sign meaning.

**Table 3:** Percentage of critical confusion errors (opposite answers) by participants for the signs for which they occurred.

Sign	Comprehension of sign meaning	Comprehension of shape-color
	**–**	**3.66**
	**7.56**	**8.22**
	3.66	**–**
	**–**	**5.65**
	4.40	**–**
	**–**	3.66
	4.40	**–**

*M* mandatory signs, *P* prohibition signs, *E* emergency signs, *W* warning signs, *F* firefighting signs.

Significant values are in bold.

### Cognitive sign features

The safety signs’ features were evaluated on five categories using a 0–100 rating scale. All of the mean ratings exceeded 60 percent, which was the highest rating related to meaningfulness (71.47). Table 4 shows the signs with the lowest and highest ratings on cognitive sign features. Although all the subjects were experienced workers, sign E12 (emergency stop) was rated as very unfamiliar (37.25). The most familiar one signed P5 (no smoking). The sign P6 (no naked flames) was perceived to be very simple and definite while the signs M3 (wear safety harness) and W16 (overhead Crane) were identified as the most complex and somewhat vague, implying that the perceived simplicity of a sign is not only related to the number of elements in the sign but may be affected by other factors such as sign concreteness or meaningfulness. The sign M3 (wear safety harness) had the lowest concreteness rating (39.28) and lowest meaningfulness rating (47.08). The E17 (fire extinguisher) sign had the highest meaningfulness rating (89.21) and semantic closeness rating (82.07), while the sign P7 (do not use this lift) had the least semantic closeness from the participants’ point of view (47.54).

**Table 4:** The lowest and the highest scored safety signs with respect to cognitive features.

Sign features	Mean ± SD	Lowest-rated signs	Highest-rated signs
Familiarity	64.15 ± 16.21		
Concreteness	60.17 ± 15.32		
Simplicity	61.22 ± 13.41		
Meaningfulness	71.47 ± 12.22		
Semantic closeness	64.35 ± 17.55		

*M* mandatory signs, *P* prohibition signs, *E* emergency signs, *W* warning signs, *F* firefighting signs, *SD* standard deviation.

### The effect of age, work experience, and education level on safety sign comprehensibility

The impact of socio-demographic factors (age, work experience, and education level) on safety signs comprehensibility was investigated. Participants' age, work experiences, and education levels were divided into five (≤ 25, 26–35, 36–45, 46–55, and ≥ 56 years), three (5–15, 16–30, and 31–45 years), and four (MS/Ph.D., bachelor’s degree, high school, less than high school) categories. Two-way analysis of variance (ANOVA) was used to analyze the difference among group means and presented in Table 5.

**Table 5:** SPSS ANOVA test of age, work experience, and education level effect on the comprehensibility of safety signs.

Safety sign	Variable	Source of variation	Sum of squares	df	Mean square	F	Sig
Mandatory signs	Age (years)	Between groups	0.344	4	0.098	1.325	0.230
		Within groups	17.312	2305	0.061		
		Total	17.656	2309			
	Experience (years)	Between groups	0.621	2	0.161	3.495	**0.028**
		Within groups	14.217	2307	0.031		
		Total	14.838	2309			
	Education level	Between groups	0.305	3	0.096	1.745	0.141
		Within groups	19.245	2306	0.521		
		Total	19.550	2309			
Prohibition signs	Age (years)	Between groups	0.421	4	0.094	2.765	**0.036**
		Within groups	17.320	2305	0.036		
		Total	17.741	2309			
	Experience (years)	Between groups	0.564	2	0.134	3.282	**0.020**
		Within groups	18.678	2307	0.041		
		Total	19.242	2309			
	Education level	Between groups	0.056	3	0.019	0.638	0.710
		Within groups	13.745	2306	0.042		
		Total	13.801	2309			
Emergency signs	Age (years)	Between groups	0.689	4	0.215	4.487	**0.000**
		Within groups	16.203	2305	0.021		
		Total	16.892	2309			
	Experience (years)	Between groups	0.766	2	0.250	7.654	**0.000**
		Within groups	14.994	2307	0.026		
		Total	15.760	2309			
	Education level	Between groups	0.287	3	0.115	2.340	0.073
		Within groups	18.559	2306	0.051		
		Total	18.864	2309			
Warning signs	Age (years)	Between groups	0.876	4	0.223	5.567	**0.000**
		Within groups	16.587	2305	0.034		
		Total	17.463	2309			
	Experience (years)	Between groups	0.588	2	0.185	4.452	**0.003**
		Within groups	16.449	2307	0.406		
		Total	17.037	2309			
	Education level	Between groups	0.273	3	0.073	2.201	0.089
		Within groups	15.774	2306	0.039		
		Total	16.047	2309			
Firefighting signs	Age (years)	Between groups	0.733	4	0.301	5.669	**0.000**
		Within groups	17.951	2305	0.028		
		Total	18.684	2309			
	Experience (years)	Between groups	0.880	2	0.365	6.102	**0.000**
		Within groups	18.245	2307	0.019		
		Total	19.125	2309			
	Education level	Between groups	0.442	3	0.120	2.275	**0.042**
		Within groups	19.345	2306	0.038		
		Total	19.787	2309			

Significant values are in bold.

Table 5 shows that the level of workers' comprehensibility of prohibition, emergency, warning, and firefighting safety signs varies significantly with the age group (p-values < 0.001). On the other hand, the level of workers' comprehensibility of mandatory signs isn’t affected by worker age (p-values = 0.230). To find out which age group has the highest effect on prohibition, emergency, warning, and firefighting safety signs comprehensibility, post-hoc tests by Bonferroni were used. For prohibition, warning, and firefighting safety signs, the age group of 36–45 years had higher comprehensibility (71.3%) than the age groups less than 25 years (53.6%) and older than 56 years (56.2%). For emergency safety signs, the age groups of 36–45 years and 46–55 years had higher comprehensibility (65.2%, and 69.4%) than the age group of fewer than 25 years (59%).

In examining the effect of education level on workers' comprehensibility, the workers' educational level had a significant effect only on the comprehensibility of firefighting signs (p-values = 0.042). Based on the Post-hoc test results by Bonferroni, participants with MS/Ph.D. and bachelor degrees had higher comprehensibility (70% and 68.4%) than the participants with an education level of less than high school (55.4%).

To find out if there are any statistically significant differences in participants' comprehensibility with working experience; a Two-way ANOVA test was used. Table 5 shows that working experience related to construction has a significant effect on the participants' comprehensibility of all safety signs (p-values < 0.001). To find out which working experience has the highest effect on safety signs' comprehensibility, post-hoc tests by Bonferroni were used. It can be concluded that participants with a working experience of 16–45 years had a higher degree of comprehensibility than those with 5–15 years of working experience (p-values < 0.001), whereas, no significant difference in the comprehensibility of safety signs were observed between workers with a working experience of 16–30 and 31–45 years (p-values < 0.001).

### Relationships between socio-demographic factors and cognitive sign features with safety sign comprehensibility

In this study, the scores of the cognitive sign features were normally distributed (Kolmogorov–Smirnov, P > 0.05). Pearson's correlation test was carried out, in each signs categories, to evaluate if there were significant correlations between the measured sign meaning and shape-color code comprehension with users' factors and cognitive sign features (see Table 6).

**Table 6:** Pearson's correlation coefficient between signs comprehension with users' factors and cognitive sign features, by signs categories.

Variable	Comprehension of sign meaning					Comprehension of shape-color				
	Ms	Ps	Es	Ws	Fs	Ms	Ps	Es	Ws	Fs
Age	0.23*	0.76**	0.81**	0.83**	0.91**	0.37**	0.71**	0.78**	0.92**	0.84**
Experience	0.81**	0.86**	0.90**	0.78**	0.85**	0.45**	0.73**	0.76**	0.80**	0.71**
Education level	–	–	–	–	–	–	–	–	–	–
Familiarity	0.67**	0.83**	0.65**	0.81**	0.71**	0.26*	0.45**	0.39**	0.55**	0.62**
Concreteness	0.51**	0.69**	0.52**	0.64**	0.32*	0.53**	0.41**	0.40**	0.36**	0.78**
Simplicity	0.61**	0.77**	0.59*	0.57**	0.74**	0.72**	0.50**	0.61**	0.32*	0.51**
Meaningfulness	0.48**	0.67**	0.55**	0.70**	0.81**	0.62**	0.58**	0.62**	0.41**	0.43**
Semantic closeness	0.40**	0.29*	0.58**	0.60**	0.80**	0.68**	0.70**	0.51**	0.77**	0.68**

*Ms* mandatory signs, *Ps* prohibition signs, *Es* emergency signs, *Ws* warning signs, *Fs* firefighting signs.

*Correlation is significant at the 0.05 level (2-tailed).

**Correlation is significant at the 0.001 level (2-tailed).

### Results of feature selection

Table 7 shows the results of various feature selection techniques. As a result of Pearson's correlation test in Table 6, it was found that only one feature namely “education level” was not correlated with safety sign comprehension (P > 0.05). However, the results of feature selection techniques in Table 7 revealed that “education level” can also be a significant feature for sign comprehensibility classification. Thus, experiments were conducted initially for all possible combinations including top 5, top 4, top 3, top 2, etc. In filter-based methods, features are arranged in decreasing order of their rank while in wrapper-based methods, the best subset of features is selected. It is found that the rank assigned to various features by different feature selection techniques is slightly different. For example, if Pearson’s correlation coefficient (P) is used as the principal criterion, “Familiarity” is considered the most reliable factor. On the other hand, if RF is used as the principal criterion, “Simplicity” is considered the most reliable factor. Similarly, “Experience” is assigned the second rank if P is used as the principal criteria while it is assigned the sixth rank if GR or IG is used as the principal criteria. It is thus concluded that relying on one principal criterion may not always result in an optimal subset of factors. An optimal subset of factors elected using one assessment measure may not be similar to that using another. The performance of various feature selection techniques is evaluated using kernel-based SVM. The corresponding results are presented and discussed in the forthcoming section.

**Table 7:** Results of various feature selection techniques.

Feature selection technique	Category of feature selection technique	Selected features in decreasing order of their rank (filter method)/ Selected subset (wrapper method)
P	Filter	Familiarity, experience, concreteness, meaningfulness, semantic closeness, simplicity, age, education level
GR	Filter	Familiarity, simplicity, concreteness, semantic closeness, meaningfulness, experience, age, education level
IG	Filter	Familiarity, simplicity, concreteness, semantic closeness, meaningfulness, experience, education level, age
1R	Filter	Familiarity, simplicity, meaningfulness, experience, concreteness, semantic closeness, age, education level
RF	Filter	Simplicity, familiarity, meaningfulness, semantic closeness, concreteness, experience, age, education level
SU	Filter	Familiarity, simplicity, concreteness, semantic closeness, experience, meaningfulness, education level, age
CFS	Wrapper	Simplicity, familiarity, experience
RRA	–	Familiarity, simplicity, experience, concreteness, meaningfulness, semantic closeness, age, education level

*P* Pearson's correlation coefficient, *GR* gain ratio, *IG* information gain, *RF* relief-F, *SU* symmetrical uncertainty, *CFS* correlation feature selection, *RRA* robust rank aggregation.

### Results of classification using kernel-based SVM

This section presents the results of different SVM classifiers with and without using the feature selection step. Six performance measures (accuracy, sensitivity, specificity, precision, F1-score, and AUC) were used for evaluation under tenfold cross-validation. Table 8 shows the performance of different SVM classifiers without using the feature selection technique (i.e. all the eight socio-demographic factors and cognitive sign features are supplied to the input of the classifier). It is found that medium Gaussian SVM outperforms other classifiers achieving the highest classification accuracy of 75.660% without using feature selection, under tenfold cross-validation. On the contrary, the course Gaussian SVM performs worst achieving the lowest classification accuracy of 54.681% under the tenfold data division protocol.

**Table 8:** Performance of various SVM-based classifiers without using feature selection under tenfold cross-validation.

Classification technique	Accuracy (%)	Sensitivity (%)	Specificity (%)	Precision (%)	F1-score	AUC
Linear SVM	73.446	68.447	77.685	88.559	0.855	0.734
Quadratic SVM	68.248	69.410	66.352	92.005	0.920	0.682
Cubic SVM	71.000	71.771	66.720	91.228	0.746	0.710
Fine Gaussian SVM	66.542	93.721	21.456	86.742	0.866	0.666
Medium Gaussian SVM	**75.660**	**75.682**	**71.456**	**95.610**	**0.948**	0.757
Course Gaussian SVM	54.681	88.235	11.230	90.118	0.785	0.547

Significant values are in bold.

Table 9 shows the performance of different SVM classifiers when the top 3 features namely, familiarity, experience, and concreteness selected by Pearson's correlation coefficient (P) are supplied as input to the classifier. It is found that the medium Gaussian SVM classifier under tenfold cross-validation outperforms others achieving a classification accuracy of 73.760%. However, fine Gaussian SVM and Linear SVM achieve a higher classification accuracy of 71.008% and 70.102%, respectively. The worst performance is demonstrated by the Cubic SVM classifier displaying the lowest classification accuracy under all data division schemes with an accuracy of 52.208%.

**Table 9:** Performance of various SVM-based classifiers using top 3 features selected by Pearson's correlation coefficient (P) feature selection evaluated by tenfold cross-validation.

Classification technique	Accuracy (%)	Sensitivity (%)	Specificity (%)	Precision (%)	F1-score	AUC
Linear SVM	70.102	70.142	55.341	88.559	0.841	0.701
Quadratic SVM	64.442	58.447	66.752	92.005	0.892	0.644
Cubic SVM	52.208	64.223	42.230	91.228	0.770	0.522
Fine Gaussian SVM	71.008	76.445	52.075	86.742	0.880	0.710
Medium Gaussian SVM	**73.760**	**74.002**	**70.125**	**95.610**	**0.922**	0.737
Course Gaussian SVM	63.506	70.142	52.36	90.118	0.807	0.635

Significant values are in bold.

Table 10 shows the performance of different SVM classifiers when the top 3 features namely, familiarity, simplicity, and concreteness selected by information gain (IG), gain ratio (GR), and symmetrical uncertainty (SU) were supplied as input to the classifier. It is found that the medium Gaussian SVM classifier outperforms others under all data division schemes with an accuracy of 75.615%. On the other hand, categories of test samples predicted by cubic SVM match least with ground truth categories resulting in its lowest classification accuracy under tenfold cross-validation.

**Table 10:** Performance of various SVM-based classifiers using top 3 features selected by the gain ratio (GR), information gain (IG), and symmetrical uncertainty (SU) feature selection evaluated by tenfold cross-validation.

Classification technique	Accuracy (%)	Sensitivity (%)	Specificity (%)	Precision (%)	F1-score	AUC
Linear SVM	70.235	72.891	68.334	82.112	0.840	0.717
Quadratic SVM	68.243	78.694	57.233	90.771	0.847	0.680
Cubic SVM	62.551	67.334	58.005	78.990	0.764	0.687
Fine Gaussian SVM	68.110	80.885	56.234	81.362	0.890	0.701
Medium Gaussian SVM	**75.615**	**85.726**	**66.235**	**93.054**	**0.910**	0.882
Course Gaussian SVM	66.773	81.335	44.337	69.770	0.791	0.699

Significant values are in bold.

Table 11 shows the performance of different classifiers when the top 3 features namely, familiarity, simplicity, and meaningfulness selected by 1R and Relief-F (RF) are supplied as input to the classifier. It is found that the medium Gaussian SVM classifier outperforms others under all data division schemes. It achieves the highest classification accuracy of 83.210% under tenfold cross-validation. On the contrary, coarse Gaussian SVM results in the lowest classification accuracy of 68.540%. It is interesting to note here that compared to all other feature combinations, the combination of familiarity, simplicity, and meaningfulness achieves the highest classification accuracy of 83.210%.

**Table 11:** Performance of various SVM-based classifiers using top 3 features selected by1R and Relief-F (RF) feature selection evaluated by tenfold cross-validation.

Classification technique	Accuracy (%)	Sensitivity (%)	Specificity (%)	Precision (%)	F1-score	AUC
Linear SVM	71.235	69.677	73.264	77.425	0.768	0.725
Quadratic SVM	79.546	85.231	72.640	88.325	0.842	0.801
Cubic SVM	79.325	85.234	71.338	86.442	0.740	0.793
Fine Gaussian SVM	77.320	89.336	63.348	79.268	0.791	0.770
Medium Gaussian SVM	**83.210**	**91.239**	**72.446**	**92.100**	**0.910**	**0.833**
Course Gaussian SVM	68.540	74.210	56.662	71.330	0.826	0.690

Significant values are in bold.

Table 12 shows the performance of different classifiers when the best subset of features selected by correlation-based wrapper feature selection (CFS) and the top 3 features selected by robust rank aggregation (RRA) is supplied as input to the classifier. The feature combination evaluated in this case is simplicity, familiarity, and experience. As in all of the previous cases, it is found that the medium Gaussian SVM classifier outperforms others under all data division schemes. It achieves the highest classification accuracy of 76.075% under tenfold cross-validation.

**Table 12:** Performance of various SVM-based classifiers using best subset selected by correlation-based wrapper feature selection (CFS) and top 3 features selected by robust rank aggregation (RRA) under tenfold cross-validation.

Classification technique	Accuracy (%)	Sensitivity (%)	Specificity (%)	Precision (%)	F1-score	AUC
Linear SVM	72.110	74.200	69.107	80.117	0.698	0.770
Quadratic SVM	68.665	76.410	63.470	84.110	0.758	0.730
Cubic SVM	62.425	66.770	55.472	73.556	0.726	0.632
Fine Gaussian SVM	70.881	80.694	60.425	74.663	0.770	0.700
Medium Gaussian SVM	**76.075**	**82.450**	**64.233**	**89.005**	**0.880**	**0.802**
Course Gaussian SVM	66.340	83.670	50.428	68.475	0.798	0.704

Significant values are in bold.

From the results of Tables 8, 9, 10, 11 and 12, it is concluded that the feature combination of familiarity, simplicity, and meaningfulness achieves the highest classification accuracy. To study and confirm the impact of these factors on safety signs comprehensibility, some other popular classifiers such as binary logistic regression (BLR), linear discriminant analysis (LDA), quadratic discriminant analysis (QDA), classification and regression tree (CART), random forest (RF), bootstrap aggregating algorithm, k-nearest neighbor (K-NN), and Adaptive Boosting were also evaluated as discussed in section "Classification" (see Table 13). It is observed that when familiarity, simplicity, and meaningfulness were used as features, the K-NN classifier achieves the highest classification accuracy of 94.369% under tenfold cross-validation. This shows that familiarity, simplicity, and meaningfulness together can have a significant impact on the prediction of safety signs comprehensibility using machine learning techniques. Other classifiers such as adaptive boosting (AdaBoost) and random forest (RF) also performed satisfactorily achieving classification accuracy of 85.260% and 83.102% under tenfold cross-validation, respectively. These results are very much comparable to those by SVM. To establish the statistical significance of improvement in classifier performance from 83.210% using medium Gaussian SVM-tenfold (see Table 11) to 94.369% using K-NN-tenfold (see Table 13), z-statistic was calculated at 95% confidence interval using approach explained in Isaac (2015) study for test concerning two proportions^60^. The z-statistic is found to be -2.204 with a p-value of less than 0.05 at a 95% confidence interval. This confirms that the improvement in classification accuracy of the K-NN classifier over the medium Gaussian SVM classifier is statistically significant.

**Table 13:** Impact of familiarity, simplicity, and meaningfulness on safety signs comprehensibility prediction using other classifiers under tenfold cross-validation.

Classification technique	Accuracy (%)	Sensitivity (%)	Specificity (%)	Precision (%)	F1-score	AUC
BLR	73.689	78.345	86.227	88.345	0.740	0.750
LDA	75.230	77.557	74.236	89.245	0.755	0.810
QDA	69.325	70.893	68.335	75.400	0.692	0.731
CART	66.880	72.368	65.448	71.664	0.670	0.684
RF	83.102	84.570	86.771	87.287	0.833	0.852
Bootstrap aggregating	79.265	80.330	79.000	83.273	0.792	0.881
K-NN	**94.369**	**95.511**	**94.276**	**95.432**	**0.950**	**0.991**
AdaBoost	85.260	88.330	84.337	86.220	0.855	0.800

*BLR* binary logistic regression, *LDA* linear discriminant analysis, *QDA* quadratic discriminant analysis, *CART* classification and regression tree, *RF* random forest, *K-NN* k-nearest neighbor, *AdaBoost* adaptive boosting.

Significant values are in bold.

Analyzing the results of Tables 8, 9, 10, 11, 12 and 13, it was found that the best combination of sensitivity, specificity, precision, F1-score, and AUC is achieved by the K-NN classifier under tenfold cross-validation when familiarity, simplicity, and meaningfulness were supplied as input to the classifier model. The values of the rest of the performance metrics such as sensitivity, specificity, precision, F1 score, and AUC were 95.511%, 94.276%, 95.432%, 0.950, and 0.991, respectively. It is also observed that, for most of the feature combinations, sensitivity is high while specificity is low.

## Discussions

In the past few decades, a large body of safety signs research has examined how to sign characteristics (such as symbol, shape, color, and incongruent information), socio-demographic factors (such as gender, age, culture, education level, work experience), and cognitive sign features impact safety signs comprehensibility^8,16,32^. These studies provide basic principles and guidelines for the design of more effective safety signs; however, the present study takes a step further using general-purpose learning algorithms to find patterns in often rich and unwieldy data that affect sign comprehension. This study assesses the safety signs comprehensibility that is used to reduce or eliminate hazards in the working environment utilizing the hierarchy of risk controls and to be part of engineering/administrative control^61,62^. This is the first study, to our knowledge, to examine the effects of socio-demographic factors and cognitive sign features on the comprehensibility performance of safety signs among construction workers using eight different feature selection techniques and various popular classifiers of machine learning (ML) approaches. In addition, supervised machine learning models presented in this study can reduce the bias existing in the workforce when making a vigilant decision on the safety signs' comprehensibility^63,64^. In this study, a database of socio-demographic factors and cognitive sign feature measurements were captured and utilized for safety sign comprehension prediction.

### User factors and cognitive sign features effects

As expected, sign comprehensibility depended on age, education level, and work experience. The present study depicted that adulthood and middle-aged construction workers have a much better perceptual performance than their older colleagues. The lower comprehensibility score in older adults (> 55 years) could be attributed to reduced attention and information-processing abilities^65^. Our results supported the previous work of Akple et al. indicating that people with a university or above education level possess better sign comprehension than the participants with an education level of less than high school^66^. Work experience, as another attribute, bore a relationship to the safety signs comprehensibility. There are investigations into construction safety signs and road warning signs that are consistent with our findings; suggesting that work experience can improve comprehension performance by increasing the frequency of encountering and familiarity with safety signs^6,67^.

In this study, the average scores of the five cognitive features were relatively close to each other but varied greatly from sign to sign. In line with the finding, Saremi et al. and Ahmadi et al. studies on pharmaceutical pictogram comprehensibility showed that the cognitive sign features differ widely from sign to sign^36,68^. For the “familiarity” feature, sign P5 (no smoking) was the most familiar sign and sign E12 (emergency stop) was rated as the least familiar sign, probably because the P5 sign is commonly seen in workplaces and public areas. For the “concreteness” rating, sign M3 (wear safety harness) and sign P6 (no naked flames) were assessed as the least- and most concrete, respectively. These results were consistent with the previous studies that concrete signs have obvious connections with the real world, while abstract signs consist mainly of shapes, arrows, and lines, and do not have such obvious connections^69,70^. Regarding sign “simplicity”, P6 (no naked flames) was perceived as the simplest one while sign W16 (overhead crane) was perceived as the most complex, implying that the perceived simplicity of a sign was related to the number of elements in the sign^71^. For the sign “meaningful”, sign E17 (fire extinguisher) and sign M3 (wear safety harness) were the most meaningful sign and the least meaningful ones, respectively.

### Determining relevant components for prediction of safety signs comprehension using machine learning paradigm

Initially, all eight features were used for classification. It was found that the top three features i.e. familiarity, simplicity, and meaningfulness selected by 1R and Relief-F (RF) achieved the highest classification accuracy among all the possible combinations. Thus, for a fair comparison between different feature selection techniques, the top three features selected by them were used for classification. It was also observed that when only the top 2 features were considered, there is a drop in classification accuracy. Hence, the top 3 features were selected for each feature selection algorithm. Results indicate that when these three features were used for classification, the accuracy of the classifier reaches 94.369% under hold out data division protocol which is even higher than that using all eight features. This further indicates that insignificant and irrelevant features may misguide the classifier model thereby deteriorating its overall performance. Among different classifiers, the K-NN classifier outperforms others under different data division protocols followed by medium Gaussian SVM. In line with the present study, Cahigas et al. stated that symbol familiarity was positively related to safety sign comprehension^72^. Saunders et al. suggest that safety management systems should use familiar signs as much as possible^3^. Also, the safety management unit should take responsibility for the appropriate placement of safety signs in different sections of construction sites and provide sign training to workers with emphasis on the adverse consequences of not giving attention to the hazards that are represented by safety signs. Regarding sign simplicity, simple signs led to a higher comprehensibility score than complex signs. This finding suggests that the extraneous decorative parts of a safety sign may confound user comprehension^67^. Lu et al. stated that good icon design should be simple and clear, especially when perceived at a distance^73^. Concerning sign meaningfulness, the comprehensibility scores were high for meaningful signs and low for meaningless signs, probably because meaningful stimuli are related to associated imagery and easily elicit meaning in one’s mind^74^.

Using the ML approach, we have shown for the first time that the comprehension of construction safety signs can be classified and assessed regardless of the prejudice that usually exists in workforces based on exposure and previous experiences. The authors wish to extend the current study and use deep learning semantic approaches in AI to quantify subjective feedback to the comprehensibility of the construction safety signs. There is hope to make the signs as general and understandable to the wide audiences without mere bias. This study has several strengths. First, it used the standard protocols for safety signs comprehensibility and cognitive signs features assessment as well as conventional ML algorithms to maximize the performance improvements in terms of results and predictions. To the best of the authors’ knowledge, no assessment is previously carried out to quantify the safety signs comprehensibility along with the evaluation of the accuracy of different ML algorithms in predicting safety signs comprehensibility and determining its most important predictors. However, the current investigation has a few limitations to note. The most significant one is the lack of transparency of ML algorithms that inherently characterize black-box ML models^75^. This means that the internal logic and inner workings of these algorithms are hidden from the user and will make a human (expert or non-expert) unable to verify, interpret and understand the reasoning of the system^76^. The current study used a series of the general ML algorithms with easy-to-understand structures and a limited number of parameters that are intrinsically transparent and can be interpreted without requiring additional explanation. As Occam’s Razor^77^ idea state the simpler model is, it may work and provide a more reliable outcome.

## Conclusions

In this study, we managed to use users’ factors and cognitive signs features for safety signs comprehensibility prediction in the construction industry using 14 machine learning models. In theory, we developed ML algorithms from three different supervised machine learning categories; namely, ensemble, neural network, and classical models. Various components of the ML paradigm like feature selection, cross-validation, classification, and performance evaluation were also implemented and examined. This study showed the role played by familiarity, simplicity, and meaningfulness in, respectively, enhancing and increasing safety sign comprehensibility. In practical terms, preventive training interventions could focus on the redesign of the actual working strategies and the adoption of engaging training methods as behavioral modeling in the use of machinery to optimize the learning of safety practices and safe behaviors. However, more study is required to confirm these findings on a larger and multi-centric database of cognitive design features among more safety signs. Large open-source databases of cognitive abilities, industrial conditions, and designing components are needed in the future to evaluate the performance of machine learning techniques in guiding the comprehensibility of the other safety signs. In the future, with a larger database, the performance of techniques used in this study can be compared with the performance of advanced classification techniques like a deep neural network. Generally, the use of a machine learning approach can be encouraged to determine which socio-demographic factors and cognitive sign features are important to predict safety signs comprehension in the construction industry. This would allow designers and practitioners to design construction safety signs based on the mental models approach to effectively convey their meaning clearly to prevent construction incidents occurrence.

## Data Availability

The datasets used and/or analyzed during the current study are available from the corresponding author upon reasonable request.
